# *Ishige okamurae* Ameliorates Methylglyoxal-Induced Nephrotoxicity via Reducing Oxidative Stress, RAGE Protein Expression, and Modulating MAPK, Nrf2/ARE Signaling Pathway in Mouse Glomerular Mesangial Cells

**DOI:** 10.3390/foods10092000

**Published:** 2021-08-26

**Authors:** Mingyeong Kim, Chiheung Cho, Changjun Lee, Bomi Ryu, Sera Kim, Jinyoung Hur, Sang-Hoon Lee

**Affiliations:** 1 Department of Food Biotechnology, University of Science and Technology, Daejeon 34113, Korea; 50034@kfri.re.kr; 2 Division of Functional Food Research, Korea Food Research Institute, 245 Nongsaengmyeong-ro, Iseo-myeon, Wanju-gun 55365, Korea; chiheungcho@kfri.re.kr (C.C.); 50040@kfri.re.kr (C.L.); k.sera@kfri.re.kr (S.K.); jyhur@kfri.re.kr (J.H.); 3 Department of Food Science and Technology, Chonbuk National University, Jeonju City 54896, Korea; 4 Marine Science Institute, Jeju National University, Jeju 63333, Korea; ryu.bomi@gmail.com

**Keywords:** *Ishige okamurae*, advanced glycation end-product, nephropathy, methylglyoxal, Nrf2/ARE pathway

## Abstract

Advanced glycation end-products (AGEs) such as methylglyoxal (MGO) play a vital role in the pathogenesis of nephropathy, a diabetic complication. In the present study, we evaluated the anti-glycation and renal protective properties of *Ishige okamurae* extract (IOE) against AGE-induced oxidative stress. HPLC analysis confirmed that bioactive phlorotannins such as diphlorethohydroxycarmalol and ishophloroglucin A are predominantly present in IOE. IOE showed strong anti-glycation activities via inhibition of AGE formation, inhibition of AGE–protein cross-linking, and breaking of AGE–protein cross-links. In addition, in vitro studies using mesangial cells demonstrated that IOE effectively suppressed intracellular reactive oxygen species production, intracellular MGO accumulation, and apoptotic cell death by MGO-induced oxidative stress, in addition to regulating the expression of proteins involved in the receptor for AGEs and nuclear factor erythroid 2-related factor 2 (Nrf2)/antioxidant response elements (ARE) signaling pathways. Therefore, IOE can serve as a natural therapeutic agent for the management of AGE-related nephropathy.

## 1. Introduction

Advanced glycation end-products (AGEs) can be generated via a glycation reaction (known as the Maillard reaction) between the carbonyl group of a reducing sugar (glucose, fructose, or ribose) in blood and the free amino group of a protein [1]. In the early stage, the condensation of glucose and amines produces an unstable Schiff base. The Schiff base is then arranged to form Amadori products. During the degradation of Amadori products, reactive carbonyl species are formed in the intermediate stage. At the advanced stage, a cross-link occurs between dicarbonyl intermediates and proteins (e.g., collagen), resulting in irreversible glycation end-products [2]. These adducts accelerate the development and progression of diabetic complications by altering the normal physiological functions of a protein upon glycation [3]. In addition, AGEs induce reactive oxygen species (ROS; e.g., superoxide (O_2_^−^), hydrogen peroxide (H_2_O_2_), peroxynitrite (ONOO^-^)), which cause intracellular oxidative stress and promote the mitochondrial apoptosis pathway by interacting with the receptor for AGEs (RAGE) [4]. The increase in AGE formation in chronic hyperglycemic conditions is a major contributor on the pathogenesis of diabetic complications including retinopathy, neuropathy, and nephropathy [5]. Therefore, inhibition of AGE formation or accumulation in the body is extensively regarded as the most effective ways for preventing or delaying AGE-related diabetic complications.

In hyperglycemic patients, there is an increase in the levels of methylglyoxal (MGO), which is one of the reactive glycation end-products and the major intermediate and precursor for AGE formation [6]. MGO is more reactive than glucose, with a stronger ability to cross-link with the arginine or lysine residues of proteins, leading to the formation of stable end-products called AGEs and subsequent activation of RAGE, which then initiates various diabetic complications [6,7]. However, MGO can directly impair cellular function independently of the AGE-RAGE pathway through intracellular reactive oxygen species (ROS) generation, inducing oxidative damage to proteins, apoptosis by inducing oxidative stress, and increasing caspase activity [5]. Several synthetic compounds, such as aminoguanidine (AG) and the thiazolium-derived compound ALT-711 (alagebrium) have been shown to inhibit the cross-linking of proteins and slow down the progression of diabetes-related complications. However, these agents were ultimately not approved for clinical use due to safety and efficacy issues during the clinical trial phase [8,9]. Therefore, the scavenging of AGEs may be a promising therapeutic approach for diabetes and its associated complications, and there is a need for the development of effective AGE-scavenging agents that are based on natural resources.

*Ishige okamurae* (*I. okamurae*) is a brown seaweed that is distributed throughout the temperate coastal region, and is generally enrich in the coast of Jeju Island, Korea. *I. okamurae* contains various natural antioxidant substances, among which phlorotannins are the most powerful water-soluble antioxidants [10,11]. Several previous studies have reported that *I. okamurae* has a variety of biological benefits associated with phlorotannins, including anti-inflammatory, anti-hypertensive, anti-bacterial, anti-tumor, anti-obesity, and free radical-scavenging activities [12,13]. *I. okamurae* acts as an inhibitor of alpha-glucosidase, which is a key enzyme in the modulation of glucose absorption, and thus enhances glucose homeostasis [14]. In addition, a phlorotannin-rich extract from *I. okamurae* has been shown to have a positive effect on genetically diabetic mice. Among the many bioactive compounds isolated from *I. okamurae*, phlorotannins have been extensively studied for their health benefits. In particular, diphlorethohydroxycarmalol (DPHC) has been considered to influence responses relevant to diabetes through the modulation of glucose-induced oxidative stress, as well as the inhibition of carbohydrate-digestive enzymes [15]. Although many previous studies have been conducted on the various biological benefits of *I. okamurae*, its preventive effects on AGE-related diabetic complications have not been investigated so far.

Brown seaweeds, which are rich in phlorotannins, are consumed for the prevention or improvement of chronic metabolic diseases due to their beneficial biological properties. On the basis of these investigations, we supposed that the phlorotannins contained in *I. okamurae* may have protective effects against AGE-induced renal damage. Therefore, in the present study, we evaluated the inhibitory effects of *I. okamurae* on AGE formation and AGE cross-links, as well as its nephroprotective effects against AGE-induced oxidative stress in mouse glomerular mesangial cells. In addition, the underlying mechanisms of action were partly elucidated using a protein expression analysis of key signaling pathways such as RAGE, MAPKs, and Nrf2/ARE.

## 2. Materials and Methods

### 2.1. Chemicals

Aminoguanidine (AG), glucose, fructose, bovine serum albumin (BSA), sodium azide, 3,3,5,5-tetramethylbenzidine (TMB) liquid substrate system for enzyme-linked immunosorbent assay (ELISA), MGO, sodium phosphate monobasic, and sodium phosphate dibasic were purchased from Sigma (St. Louis, MO, USA). Dulbecco’s modified Eagle’s medium/F-12 Nutrient Mixture (DMEM/F-12; Ham, 3:1 mixture) was obtained from Welgene Inc. (Daegu, Korea). Fetal bovine serum (FBS), hydroxyethyl-piperazineethane-sulfonic acid (HEPES), penicillin/streptomycin solution, and collagen I-coated plates were purchased from Gibco (Rockville, MD, USA). PRO-PREP™ Protein Extraction Solution, the OxiSelect™ Methylglyoxal (MG) Competitive ELISA Kit, and the RAGE antibody were purchased from iNtRON Biotechnology (Seongnam, Korea), Cell Biolabs Inc. (San Diego, CA, USA), and Merck Millipore (Billerica, MA, USA), respectively. Antibodies against catalase (CAT), heme oxygenase 1 (HO-1), NAD(P)H: quinone oxidoreductase 1 (NQO1), extracellular signal-regulated kinases (ERK), phospho-ERK (p-ERK), c-Jun N-terminal kinase (JNK), phosphor-JNK (p-JNK), p38, phosphor-p38 (p-p38), and superoxide dismutase 1 (SOD1) were purchased from Cell Signaling Technology (Danvers, MA, USA). β-actin and nuclear factor erythroid 2-related factor (Nrf2) antibodies were obtained from Santa Cruz Biotechnology (Santa Cruz, CA, USA). The receptor for advanced glycation end-products (RAGE) antibody was obtained from Merck Millipore (Billerica, MA, USA).

### 2.2. Sample Preparation

In brief, *Ishige okamurae* (*I. okamurae*) was collected from Jeju Island, South Korea. *I. okamurae* was washed with tap water to remove salt, sand, and epiphytes attached to the surface. Next, *I. okamurae* was lyophilized and ground to obtain a dry powder. Dried *I. okamura* powder was extracted in 50% ethanol under reflux conditions. The extract was concentrated and freeze-dried (final yield: 87 g). *I. okamurae* extracts (IOEs; kindly provided by Prof. Y.J. Jeon) were sealed and stored at −20 °C prior to use.

### 2.3. Identification of Phlorotannins in I. okamurae Using HPLC

Chromatographic analyses were conducted on an Acquity™ Arc equipped with a 2998 PDA detector and an Acquity™ QDa detector system (Waters Corporation, Beverly, MA, USA). The phlorotannin compounds in *I. okamurae* extract were analyzed using a Poroshell 120 EC-C18 column (4 µm, 4.6 mm × 100 mm; Agilent(Santa Clara, CA, USA)). The mobile phase consisted of (A) 0.1% formic acid in water and (B) acetonitrile containing 0.1% formic acid. The HPLC elution condition consisted of an isocratic system of 32% B at a flow rate of 0.4 mL/min and an injected volume of 10 µL.

### 2.4. Inhibitory Effect of I. okamurae on AGE Formation

The inhibiting ability of IOE on AGE formation via the Maillard reaction was evaluated according to the method described by Do et al. [16], with slight modifications. BSA (10 mg/mL), glucose (100 mM) and fructose (100 mM) were incubated in phosphate-buffered saline (PBS, pH 7.4 with 0.02% sodium azide), in the presence or absence of AG (0.5 mM) or IOE (5, 20, and 100 μg/mL) at 37 °C for 7 day. AGE formation level was measured at an excitation wavelength of 350 nm and an emission wavelength of 450 nm, using a microplate reader (Molecular Devices, Sunnyvale, CA, USA). The AGE formation level was expressed as a percentage (%) decrease in the fluorescence intensity of the control (without test samples).

### 2.5. AGE Cross-Link Breaking Effect of I. okamurae

The effect of IOE on breaking AGE-BSA-induced cross-linking with collagen was evaluated according to the method of Kim et al. [17] with slight modifications. To confirm the IOE-induced breaking activity of AGE-BSA-induced cross-linking of collagen, 1 mg/mL of AGE-BSA was added to collagen-coated 96-well plates and incubated at 37 °C for 4 h, following which the AGE-BSA complexes with collagen were incubated in either the presence or absence of ALT-711 or IOE at 37 °C for 18 h. The unattached AGE-BSA was washed with 0.05% PBST. Breaking levels were detected using TMB substrate. Breaking of AGE-induced cross-links was expressed in terms of percentage decrease in optical density.

### 2.6. AGE Cross-Link Inhibitory Effect of I. okamurae

The inhibitory effect of IOE on AGE-BSA-induced cross-linking to collagen was evaluated according to the method described by Do et al. [18], with slight modifications. To confirm the inhibitory effect of IOE on AGE-induced cross-linking to collagen, 1 mg/mL of AGE-BSA was incubated with AG (0.5 mM) or IOE (5, 20, and 100 μg/mL) on collagen-coated 96-well plates at 37 °C for 18 h. The inhibitory ability of IOE was detected using TMB as a substrate. Inhibition of AGE-induced cross-linking was expressed in terms of percentage decrease in optical density.

### 2.7. Cell Culture

Mouse glomerular mesangial cells were cultured in a 3:1 mixture of DMEM/F-12 medium containing 5% FBS, 14mM HEPES, 100 U/mL penicillin, and 100 μg/mL streptomycin at 37 °C in 5% CO_2_ and 95% air.

### 2.8. Nephroprotective Effect of I. okamurae

The nephroprotective effects of IOE were determined using a 3-(4,5-dimethylthiazol-2-yl)-2,5-diphenyl tetrazolium bromide (MTT) reduction assay. Mesangial cells were seeded at a density of 3.0 × 10^4^ cells per well in a 96-well plate with 100 μL of DMEM/F12 medium containing 5% FBS and incubated for 6 h before sample treatment. The supernatant was removed and the cells were pretreated with various concentrations of IOE for 1 h, followed by treatment with MGO for 24 h. After removing the medium with samples, 0.5 mg/mL MTT reagent was added to the cells and incubated for 3 h at 37 °C. The resulting formazan product was dissolved in dimethyl sulfoxide. The number of viable cells was determined by measuring the absorbance at 570 nm (detection wavelength) and 630 nm (reference wavelength) using a microplate reader (Infinite M200; Tecan Austria GmbH, Grödig, Austria). Viability was expressed in terms of percentage (%) of absorbance of the control cells (without MGO and IOE).

### 2.9. Intracellular ROS Scavenging Activity of I. okamurae

The intracellular antioxidant capacity was determined using a fluorescent probe (2′,7′-dichlorofluorescin diacetate or DCFH-DA). Mouse glomerular mesangial cells were seeded at a density of 3.0 × 10^4^ cells per well in a 96-well plate with 100 μL of DMEM/F12 medium containing 5% FBS and incubated for 6 h. The supernatant was subsequently removed, following which the cells were treated with various concentrations of IOE for 1 h, and then treated with MGO for 2 h. The cells were then washed twice with Hank’s balanced salt solution (HBSS) and incubated with 10 μM DCFH-DA in HBSS for 30 min. Fluorescence was measured using an Infinite M200 microplate reader. ROS production levels in the cells were measured at excitation and emission wavelengths of 485 and 530 nm, respectively. The degree of intracellular ROS levels was expressed as percentage (%) of the non-treated group (without MGO and IOE).

### 2.10. MGO-Derived AGE Concentration

The ability of IOE to inhibit intracellular AGE accumulation was evaluated by measuring the concentrations of MGO-cross-linked proteins using the OxiSelect™ Methylglyoxal (MG) Competitive ELISA Kit (Cell biolabs, San Diego, CA, USA). Mesangial cells were seeded at a density of 1.0 × 10^6^ cells per 6-well plate and treated with MGO, with or without IOE, for 24 h. The cells were then collected and lysed to measure the MGO-derived AGE concentrations using an ELISA kit, by measuring the absorbance at 450 nm using an Infinite M200 microplate reader.

### 2.11. Apoptosis Analysis

Induction of apoptosis was measured using the Muse™ Annexin V & Dead Cell Kit (Luminex, Austin, TX, USA). Mesangial cells were seeded at a density of 1.0 × 10^6^ cells per 6-well plate with 2 mL of DMEM/F12 medium containing 5% FBS and incubated for 6 h. Cells were pretreated with various concentrations of IOE for 1 h. Following that, MGO was added to the wells to induce apoptosis for 23 h. After removing the medium with samples and MGO, the cells were washed with cold HBSS and diluted to a density of 500 cells/μL prior to staining. Cells were resuspended in DMEM/F12 medium, added to the Muse™ Annexin V & Dead Cell Reagent, and then incubated for 20 min at room temperature in the dark. The assay results were measured using a Muse™ Cell Analyzer (Merck Millipore, Sydney, Australia). Results have been expressed in terms of percentage of total apoptotic cells (early and late apoptotic cells), percentage of live cells, and percentage of dead cells.

### 2.12. Western Blotting

The pretreated cells were scraped, and intracellular proteins were extracted using PRO-PREP™ Protein Extraction Solution for Cell/Tissue containing 1% protease and phosphatase (Thermo Fisher Scientific, Rockford, IL, USA). First, the protein contents were measured using a DC Protein Assay (Bio-Rad, Hercules, CA, USA), following which the protein content of each group was diluted to the same concentration. After being bathed in a metal bath at 100 °C for 5 min, the protein was cooled to room temperature and used as a backup. The remaining protein samples were stored at −80 °C until use. The diluted protein samples were subjected to electrophoresis at 200V using Any kD™ Mini-PROTEAN^®^ TGX Stain-Free™ Gels, following which the separated proteins were transferred to the Trans-Blot^®^ Turbo™ RTA Transfer Kit polyvinylidene difluoride membrane (Bio-Rad, Hercules, CA, USA). The membranes were blocked with 5% skim milk in Tris-buffered saline with 0.1% Tween-20 for 1 h. The membranes were then incubated with primary antibodies (used at a dilution of 1:1000) with weak shaking overnight at 4 °C. After incubation, the membranes were incubated with HRP-labeled goat anti-rabbit IgG or goat anti-mouse IgG (used at a dilution of 1:5000) for 1 h at room temperature. Finally, the membrane was exposed to WesternSure^®^ Chemiluminescent Substrate (LI-COR, Lincoln, NE, USA), and luminescence was detected using ChemiDoc™ (Bio-Rad, Hercules, CA, USA).

### 2.13. Statistical Analysis

All experiments were independently performed in triplicate, and data are presented as mean ± standard deviation (*n* = 3). To assess the significant differences in the mean values, one-way analysis of variance was performed followed by the Tukey’s multiple comparisons test (*p* < 0.05) using GraphPad Prism ver. 9 (GraphPad Software, Inc., San Diego, CA, USA).

## 3. Results

### 3.1. Identification of Phlorotannins in I. okamurae

**Figure 1:**
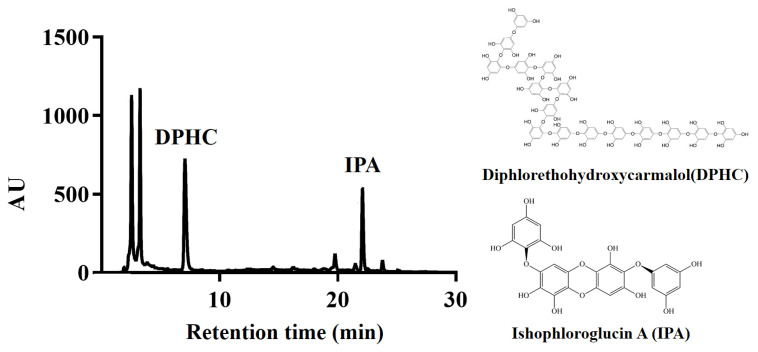
HPLC chromatogram of *Ishige okamurae* extract. It was confirmed that *Ishige okamurae* 50% ethanol (*v*/*v*) extract contained two phlorotannin compounds diphlorethohydroxycarmalol (DPHC) and ishophloroglucin A (IPA), which were identified.

Marine algae produce metabolites necessary for the growth and propagation of the organism involved with developmental stages and further defense responses against external environmental changes. Enabling the nutraceutical use of *I. okamurae* requires the identification and quantification of its chemical components and biological activity. DPHC and IPA were identified as the predominant bimodal peaks in the chromatogram obtained for *I. okamurae* (Figure 1). The mass spectrum of each phlorotannin was determined systemically by comparison with previous studies on the identification of *I. okamurae* composition [19,20]; which is phlorotannins in the form of organic polymers of phloroglucinol (1,3,5-trihydroxybenzene), DPHC (512.06 g/mol, 7.1 min), and IPA (1986.26 g/mol, 22.2 min). The concentrations of DPHC and IPA in the *I. okamurae* used in this study were 2.61 ± 0.27% and 2.43 ± 0.44%, respectively.

### 3.2. Anti-Glycation Abilities of I. okamurae 

**Figure 2:**
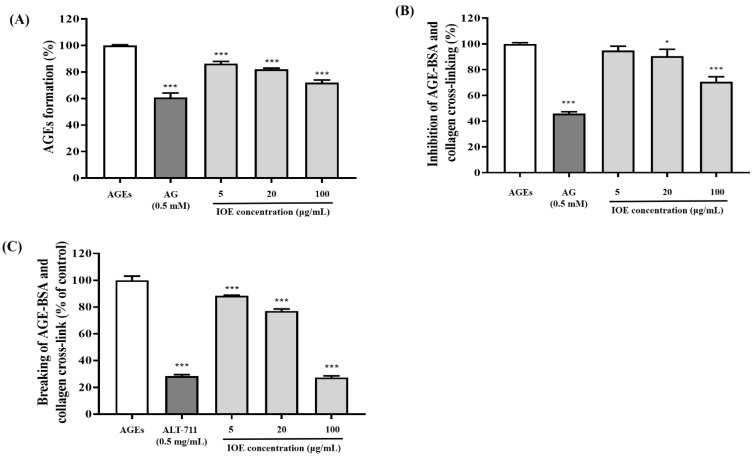
Effects of the *Ishige okamurae* extract on AGE-induced glycation reaction in vitro. Inhibition of AGE formation (**A**), inhibition of AGEs-BSA and collagen cross-link formation (**B**), and breaking of AGEs-BSA and collagen cross-links (**C**). Bar values are expressed as mean ± standard deviation (*n* = 6) (* *p* < 0.05 and *** *p* < 0.001 vs. AGEs).

In our study, we evaluated the in vitro anti-glycation property of IOE (Figure 2). An AGE formation assay was performed to assess the inhibitory ability of IOE on AGE formation. As shown in Figure 2A, incubation with BSA-glucose and -fructose induced AGE formation. However, incubation with IOE (5, 20, and 100 μg/mL) significantly (*** *p* < 0.001) reduced AGE formation. In addition, the inhibitory effect of IOE on AGE formation was similar to that of 0.5 mM AG, a synthetic compound developed to prevent diabetes complications.

ELISA was performed to evaluate the inhibitory ability of IOE on AGE-BSA and collagen cross-linking. The AGE-BSA mixture considerably increased cross-linking between the AGEs and collagen, but treatment with IOE significantly (* *p* < 0.05 or *** *p* < 0.001) inhibited the formation of cross-links between the AGEs and collagen in a dose-dependent manner (Figure 2B). In particular, IOE treatment, at a concentration of 100 μg/mL displayed a cross-linking inhibitory effect identical to that of AG, which was used as a positive control.

The effect of IOE on breaking the cross-links formed between AGEs and collagen was evaluated using ELISA (Figure 2C). IOE treatment was confirmed to effectively cleave the cross-links formed between AGEs and collagen, at a level comparable to that of ALT-711, which is known to serve as a representative cross-link breaker. Overall, upon evaluating the anti-glycation property of IOE through various in vitro models, it was confirmed that IOE treatment not only inhibited AGE formation, but also inhibited the formation of, and destroyed the cross-links formed between, AGEs and collagen.

### 3.3. Protective Effects of I. okamurae against MGO-Induced Renal Damage in Mesangial Cells 

**Figure 3:**
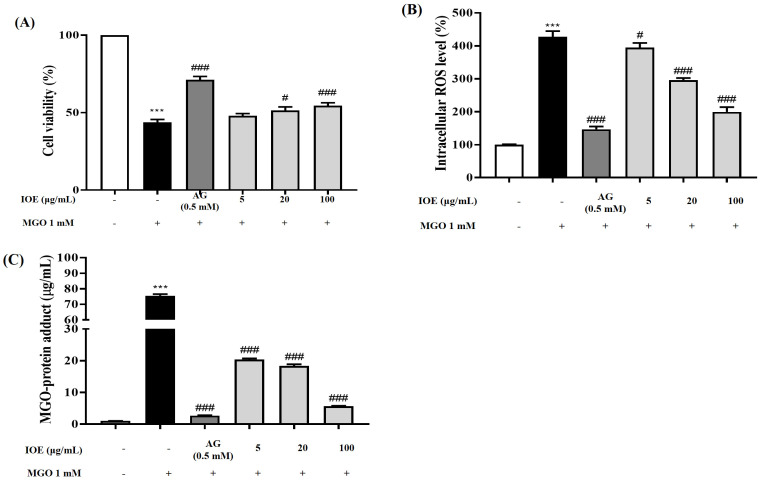
Protective effects of IOE on MGO-induced renal damage. Protective effect (**A**), intracellular antioxidant capacity (**B**) and MGO−cross−linked protein accumulation inhibitory effect (**C**)of IOE on MGO−induced renal damage in mouse glomerular mesangial cells. A decrease in cell viability to lower than 80% compared to non−treated group was considered to be cytotoxic. Each experiment was performed three times independently. Bar values are presented as mean ± standard deviation (*n* = 6). (*** *p* < 0.001 vs. normal group and # *p* < 0.05, ### *p* < 0.001 vs. MGO−treated group).

*I. okamurae* has strong antioxidant effects in vitro. Thus, we evaluated whether IOE can relieve oxidative stress caused upon MGO exposure. First, we examined the cytotoxicity of IOE using an MTT assay. A decrease in cell viability to lower than 80% compared to the non-treated group was considered cytotoxic. All the concentrations (5, 20, and 100 μg/mL) of IOE tested in this study were found to be non-cytotoxic to mesangial cells (data not shown). MGO, which is one of the major contributory elements of diabetes complications, can induce renal damage in various biological and pathological processes, resulting in cell death. The cell viability of mesangial cells exposed to MGO was lower (approximately 67% decrease) than control cells (non-treated group) (100%) (Figure 3A). Treatment with IOE significantly (# *p* < 0.05 or ### *p* < 0.001) increased cell viability, as compared to the MGO-treated group, in a dose-dependent manner. Pre-treatment with 100 μg/mL of IOE increased mesangial cell viability by up to approximately 55%, and exhibited the same level of nephroprotective effects as 0.5 mM AG, which was used as a positive control. Thus, it was confirmed that IOE protected mouse glomerular mesangial cells from MGO-induced oxidative stress at all the treatment concentrations, which may help treat diabetic complications such as nephropathy.

The intracellular antioxidant capacity of IOE was confirmed by performing a DCFH-DA assay to confirm the ability of IOE to reduce MGO-induced intracellular ROS production in mouse glomerular mesangial cells (Figure 3B). When mesangial cells were exposed to 0.5 mM MGO (used as oxidative stress) for 1 h, there was a significant (*** *p* < 0.001) increase in the ROS production levels, up to approximately 427%, as compared to the non-treated control (100%). AG (0.5 mM; used as positive control) reduced MGO-induced intracellular ROS production level to approximately 147%. Pre-treatment of mesangial cells with IOE caused a significant decrease in intracellular ROS production in a dose-dependent manner (# *p* < 0.05 or (### *p* < 0.001). The intracellular antioxidant capacity of 100 μg/mL IOE suppressed ROS production by up to 199%. The results of this in vitro study showed that pretreatment with IOE had a remarkable renoprotective effect, by inhibiting MGO-induced oxidative stress.

MGO treatment is known to increase intracellular MGO accumulation and MGO-derived AGE concentration, resulting in MGO-induced oxidative cell damage and cell apoptosis in diverse cell lines. In this study, it was confirmed that MGO treatment significantly increased MGO accumulation in mesangial cells, while IOE treatment significantly reduced intracellular MGO-protein adduct concentration (### *p* < 0.001) (Figure 3C).

### 3.4. Preventive Effects of I. okamurae against MGO-Induced Apoptotic Cell Death 

**Figure 4:**
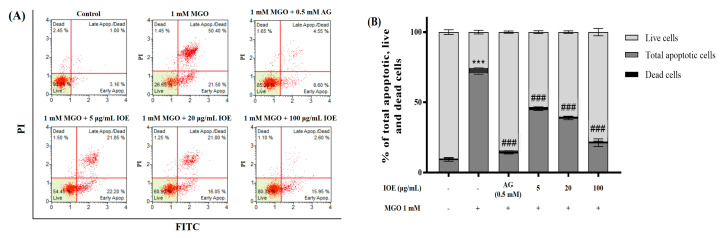
Protective effects of IOE on MGO−induced apoptotic cell death. Preventive effect of IOE on MGO-induced apoptosis in mouse glomerular mesangial cells. Dot plots for flow cytometric analysis of apoptotic cells. Annexin V−positive cells were assigned to the upper−right (late stage apoptotic cells) and lower−right (early stage apoptotic cells) quadrants, respectively. Dead cells are presented in the upper−left in the dot plots. Living cells without signs of apoptosis in the lower−left quadrant were negative for both Annexin V & PI staining (**A**). Bar graph showing the percentage of live, apoptotic (early and late apoptotic), and dead cells determined using the Muse™ Annexin V & Dead Cell Kit (**B**). Each experiment was performed three times independently. Bar values are presented as mean ± standard deviation (*n* = 3) (*** *p* < 0.001 vs. normal group and ### *p* < 0.001 vs. MGO−treated group).

The apoptotic cascade of cells is known to induce the cleavage of protein substrates and apoptotic cell death. Quantitative measurement of apoptotic cell death was carried out using flow cytometry with the Muse™ Annexin V & Dead Cell Kit. Apoptotic cells were stained with annexin V (FITC), whereas necrotic cells were stained with PI (Figure 4A). Treatment of mesangial cells with MGO significantly (*** *p* < 0.001) reduced the percentage of live cells from 90.0% to 25.6% and increased the percentage of total apoptotic cells from 9.4% to 71.1%. However, when mesangial cells were treated with IOE, apoptotic cell death was significantly suppressed (### *p* < 0.001), up to 21.2% at an IOE concentration of 100 μg/mL (Figure 4B). In addition, it was confirmed that IOE treatment suppressed the MGO-induced apoptotic cascade of mesangial cells, thereby maintaining a level of live cells similar to that seen in the normal group (Figure 4B). These results showed that IOE has the potential to prevent AGE-related renal damage by effectively inhibiting MGO-induced cell death, intracellular ROS production, intracellular MGO-protein adduct accumulation, and apoptotic cell death.

### 3.5. Effects of I. okamurae on RAGE Expression in Mesangial Cells 

**Figure 5:**
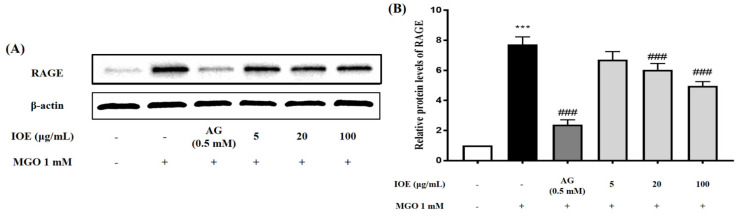
Effects of IOE on RAGE protein expression in MGO−induced mouse glomerular mesangial cells. Cells were pretreated with IOE for 1 h, followed by incubation with 1 mM MGO for 24 h. The protein expression levels of RAGE were measured using Western blots (**A**). RAGE band intensity; β-actin was used as an internal control (**B**). Each experiment was performed three times independently. Bar values are presented as mean ± standard deviation (*n* = 3) (*** *p* < 0.001 vs. normal group and ### *p* < 0.001 vs. MGO−treated group).

The increased formation of AGEs or accumulation of AGEs is a major contributor in the development of diabetic complications such as nephropathy. In addition, AGEs such as MGO are known to be directly involved in ROS production as well as transducing intracellular signals through RAGEs. Therefore, the effect of IOE on RAGE protein expression was evaluated using Western blots in mesangial cells (Figure 5A). MGO treatment increased RAGE protein expression by approximately 4-fold, as compared to normal cells (non-treated group). On the other hand, IOE treatment significantly (### *p* < 0.001) suppressed MGO-induced RAGE protein expression (Figure 5B). In addition, IOE not only effectively suppressed RAGE protein expression at all the tested concentrations, but also exhibited an inhibitory effect on RAGE protein expression that corresponded with that of AG, which was used as a positive control.

### 3.6. Effects of I. okamurae on Nrf2/ARE Pathway 

**Figure 6:**
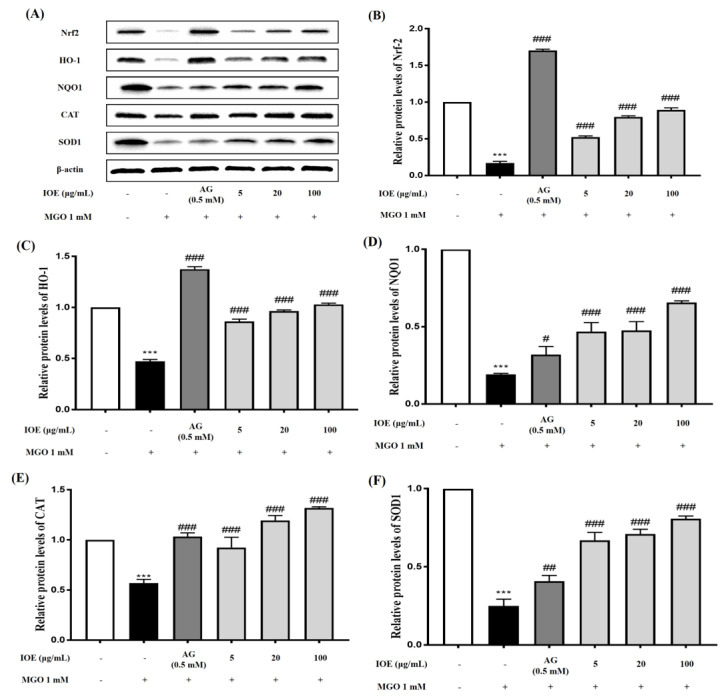
Effect of IOE on expression of Nrf2/ARE in MGO-induced mouse glomerular mesangial cells. Cells were pretreated with IOE for 1 h, followed by incubation with 1 mM MGO for 24 h. The protein expression levels of Nrf2, HO−1, NQO1, CAT, and SOD1 were measured using Western blot (**A**). Nrf2, HO-1, NQO1, CAT, and SOD1 band intensity; β-actin was used as an internal control (**B**–**F**). Each experiment was performed three times independently. Bar values are presented as mean ± standard deviation (*n* = 3) (*** *p* < 0.001 vs. normal group and *# p* < 0.05, ## *p* < 0.01, and ### *p* < 0.001 vs. MGO−treated group).

To assess the preventive ability of IOE against MGO-induced renal damage, the Nrf2/ARE signaling pathway was investigated by Western blotting (Figure 6A). Nrf2 protein expression was significantly lower in MGO-treated cells compared to the control group (Figure 6B). The expression of Nrf2 protein was significantly increased in 2 μg/mL IOE-treated cells and was similar to that of AG-treated cells. Nrf2 protein expression increased in a concentration-dependent manner. Furthermore, MGO treatment significantly reduced the expression of antioxidant enzymes such as HO-1, NQO1, CAT, and SOD1 in mesangial cells (*** *p* < 0.001), whereas IOE treatment significantly improved (### *p* < 0.001) (Figure 6C–F). These results indicate that IOE can protect mesangial cells from MGO-induced oxidative stress by increasing the expression of proteins involved in the Nrf2/ARE pathway.

### 3.7. Effects of I. okamurae on MAPK Phosphorylation 

**Figure 7:**
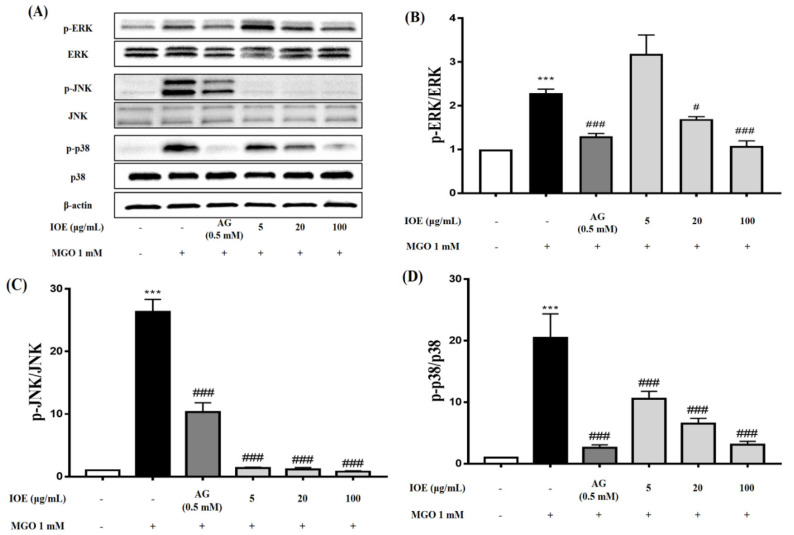
Effect of IOE on MAPK signaling pathways in MGO−induced mouse glomerular mesangial cells. Cells were pretreated with IOE for 1 h, followed by incubation with 1 mM MGO for 24 h. The protein expression levels of MAPKs (p−ERK, ERK, p−JNK, JNK, p−p38, and p38) were examined using Western blot (**A**). p−ERK/ERK, p−JNK/JNK, and p−p38/p38 ratios (**B**–**D**). Data are presented as mean ± standard deviation (*n* = 3) (*** *p* < 0.001 vs. normal group and # *p* < 0.05, and ### *p* < 0.001 vs. MGO−treated group).

Phosphorylation-induced activation of MAPK is a crucial stage in MGO-induced apoptosis. As shown in Figure 7A, the phosphorylation of ERK, JNK, and p38 in MGO-treated mesangial cells was analyzed by Western blotting using the following antibodies: p-ERK, ERK, p-JNK, JNK, p-p38, and p38. Treatment of mesangial cells with MGO caused a significant increase in the phosphorylation of ERK, JNK, and p38. Conversely, IOE pretreatment suppressed their phosphorylation (Figure 7B–D).

## 4. Discussion

Under hyperglycemic conditions, the carbonyl group of sugar and the free amino group of biological proteins become AGEs. During glycation, reducing sugars are dicarbonyl compounds, including MGO, glyoxal, and 3-deoxyclucosone. Of these, MGO is the most reactive compound, and well known for its glycation potency [7]. MGO has been shown to induce aging, retinopathy, cardiovascular diseases, and renal dysfunction via dicarbonyl stress in vivo [6]. In addition, elevated levels of MGO in Drosophila have been shown to induce fatty acid synthase, hyperglycemia, MGO-adducts, and insulin resistance [21]. Moreover, accumulation of AGEs such as MGO in the tissues is implicated in AGE-related diabetes complications because it alters enzyme activity, reduces ligand binding, modifies protein half-life, and alters immunogenicity [22]. Therefore, inhibiting the accumulation of AGEs, suppressing the production of AGEs, inhibiting/breaking cross-links between AGEs and proteins, or reducing AGE-induced oxidative stress is believed to be an effective strategy to prevent and delay AGE-related diabetic complications.

*I. okamurae*, an edible brown alga, is known to be effective in ameliorating blood glucose levels and insulin resistance, as well as, exhibiting strong antioxidant activity [13,23]. Several previous studies have reported that natural plant resources, especially those with potent antioxidant compounds appear to play a vital action in the improvement of disorders involving oxidative stress-related diabetes mellitus [24]. Additionally, DPHC and IPA, which are representative compounds derived from *I. okamurae*, are known to suppress elevated blood glucose levels after a meal and improve glucose homeostasis [13,15]. Therefore, we determined the DPHC and IPA contents of *I. okamurae* using HPLC analysis (Figure 1). In our study, IOE showed anti-glycation properties by inhibiting AGE formation and breaking/inhibiting AGEs-collagen cross-links (Figure 2). These results indicated that *I. okamurae* inhibits AGEs through a variety of mechanisms, suggesting that it is effective in preventing and delaying AGE-related diabetes complications.

Chronic hyperglycemic conditions further accelerate this reaction and increase the accumulation of AGEs in body tissues. AGE accumulation has been implicated in the development of insulin resistance and AGE-related diabetic complications. MGO treatment increases intracellular MGO concentrations and MGO-derived AGE concentrations, subsequently causing cellular damage and apoptotic cell death by oxidative stress in diverse cells [25]. In addition, MGO, which is a reactive dicarbonyl compound of AGEs, increases intracellular oxidative stress and induces apoptosis [7]. In the present study, *I. okamurae* treatment exhibited protective effects against MGO-induced renal damage and intracellular ROS accumulation in mouse kidney mesangial cells (Figure 3). In addition, *I. okamurae* not only significantly reduced intracellular MGO accumulation, but also inhibited apoptotic cell death mediated by MGO-induced oxidative stress (Figure 4). Therefore, our results demonstrated that IOE protects against MGO-induced cytotoxicity by downregulating MGO-induced ROS generation, MGO-cross-linked protein concentration, and apoptotic cell death.

Our results indicated that IOE significantly reduced the level of apoptosis caused by MGO, but the mechanism of action was not clear. We hypothesized that certain signaling pathways activated by MGO may be involved in the mechanism of action. To determine whether RAGE plays an important role in MGO-induced cell dysfunction, we first measured the protein expression of the AGE receptor RAGE (Figure 5). RAGE expression increased upon MGO treatment, but significantly decreased upon IOE treatment. The interaction of AGEs with RAGE enhances oxidative stress through ROS production by NADPH oxidases inside the mitochondria [26].

We hypothesized that IOE would suppress MGO-induced toxicity by causing a change in the Nrf2 signaling pathway while reducing RAGE expression. Nrf2 is an essential regulator of ARE-mediated induction, including the regulation of antioxidant enzymes such as SOD, NQO1, and catalase. Several studies have reported that Nrf2 regulates AGE-induced inflammatory cytokine concentrations and oxidative stress [27]. The transcription factor Nrf2 normally forms a complex with the cytoskeleton-associated protein Keap1 in the cytoplasm. When cells are exposed to oxidative stress, the Nrf2/Keap1 interaction is broken and free Nrf2 translocates into the nucleus to activate ARE-dependent genes [28,29]. To determine whether IOE can activate Nrf2 under MGO treatment, we performed a Western blot with MGO-treated samples. In our study, MGO treatment significantly reduced Nrf2 protein expression and its downstream pathway molecules (Figure 6). However, IOE treatment significantly increased Nrf2 protein expression and the expression of antioxidant enzymes such as HO-1, NQO1, CAT, and SOD1. Therefore, our results suggest that IOE has an inhibitory effect on MGO-induced cytotoxicity by regulating the Nrf2 signaling pathway and reducing ROS production in renal cells.

MAPKs play an important role in cell differentiation and apoptosis. ERK, JNK, and p38 are major proteins in the MAPK group. ERK is associated with the proliferation and progression of certain cellular systems, but JNK, p38, and ERK are also associated with apoptosis [30,31]. In this study, we observed that pretreatment with IOE most dramatically inhibited the activation of ERK, JNK, and p38 (Figure 7). Inhibition of apoptosis by IOE was accompanied by inhibition of MAPK activation, suggesting that IOE can regulate the MAPK signaling pathway in MGO-treated intermediate cells. MAPKs can be activated independently and are involved in cell death. In recent years, it has been suggested that MGO-induced cytotoxicity is associated with the activation of members of the MAPK family, including ERK, JNK, and p38 [25]. Therefore, our results suggest that IOE has an inhibitory effect on MGO-induced cytotoxicity by regulating the Nrf2/ARE pathway and reducing ROS generation in renal cells.

An increase in the levels or accumulation of intracellular AGEs has been reported as one of the immediate causes of diabetes development and its related complications. Therefore, many researchers have attempted to prevent/improve/treat AGE-related diabetes and complications using natural resources or active compounds derived from them, as these present a low risk of side effects. Our results showed that *I. okamurae*, an edible brown alga, effectively inhibited various processes related to the production and accumulation of AGEs through actions such as inhibition of AGE formation, inhibition/breaking of AGE-protein cross-linking. In addition, it was demonstrated that *I. okamurae* exerts nephroprotective effects by reducing MGO-induced ROS generation and regulating the expression of proteins involved in the RAGE and Nrf2/ARE pathways in mesangial cells. Therefore, *I. okamurae* is considered a very useful natural resource with the potential to prevent and treat diabetic complications such as AGE-related diabetic nephropathy. However, since various physiologically active compounds exist in *I. okamurae*, additional studies using single phlorotannin compounds contained in *I. okamurae* are needed to clarify its efficacy. In addition, in order to verify the efficacy of the phlorotannin compound contained in *I. okamurae* for AGE-induced nephropathy, an in vivo experiment using an animal model is necessary.

## 5. Conclusions

In conclusion, our results clearly indicated that *I. okamurae* possesses anti-glycation activity. In addition, it was demonstrated that *I. okamurae* effectively protects renal cells by reducing MGO-induced ROS generation and oxidative stress, as well as increasing the expression of proteins involved in antioxidant defense mechanisms. Consequently, *I. okamurae* may be used as a potential natural therapeutic agent for the management of AGE-related diabetic complications as an AGE inhibitor and breaker. In particular, *I. okamurae* containing phlorotannins such as IPA and DPHC should be considered to be effective in preventing AGE-related nephropathy.

## Abbreviations

IOE	*Ishige okamuare* extract
AGEs	advanced glycation end-products
RAGE	receptor for advanced glycation end-products
MGO	methylglyoxal
DPHC	diphlorethohydroxycarmalol
IPA	ishophloroglucin A
ARE	antioxidant response elements
ROS	reactive oxygen species
AG	aminoguanidine
ALT-711	alagebrium
BSA	bovine serum albumin
DCFH-DA	2,7-dichlorodi-hydrofluorescein diacetate
TMB	tetramethylbenzidine
PBST	phosphate buffered saline with Tween^®^ 20
CAT	catalase
ERK	extracellular signal-regulated kinase
JNK	c-Jun N-terminal kinase
HO-1	heme oxygenase-1
NQO1	NAD(P)H Quinone Oxidoreductase 1
Nrf2	nuclear factor erythroid 2-related factor 2
SOD1	superoxide dismutase
